# Disseminated multidrug-resistant *Salmonella enterica* serovar Dublin infection associated with plasmid-borne CMY-2 gene in a patient with travel to a farm in Mexico

**DOI:** 10.1007/s10096-026-05536-y

**Published:** 2026-05-12

**Authors:** Mackenzie E. Collins, Lilian Fung, Timothy F. Brewer, Shangxin Yang

**Affiliations:** 1 Department of Pathology and Laboratory Medicine, David Geffen School of Medicine, University of California, Los Angeles, CA USA; 2 Division of Infectious Diseases, David Geffen School of Medicine, University of California, Los Angeles, CA USA; 3 Department of Epidemiology, Fielding School of Public Health, University of California, Los Angeles, CA USA; 4 UCLA Clinical Microbiology Laboratory, 11633 San Vicente Blvd, Los Angeles, CA 90049 USA

**Keywords:** Zoonotic infection, Salmonellosis, Plasmid-borne AmpC, Genomic surveillance, Non-typhoidal *Salmonella*, Dissemination

## Abstract

Disseminated *Salmonella enterica* infections represent a severe form of non-typhoidal salmonellosis increasingly complicated by antimicrobial resistance. We describe a case of disseminated non-typhoidal *Salmonella* in a man presenting with septic shock concerning for a thoracoabdominal endovascular graft infection with involvement of the urinary and respiratory tracts and thoracic skeleton. Hybrid whole-genome sequencing identified *Salmonella enterica* serovar Dublin and two significant plasmids, an IncC multidrug resistance plasmid harboring *bla*_CMY−2_, *bla*_TEM−206_, *tet*(*A*), and *sul2* and an IncFII(S)/IncX1 virulence plasmid containing a *spvA*-*D*/*spvR* operon associated with systemic infection. This case highlights a potential food-borne or zoonotic acquisition of invasive, plasmid-mediated AmpC-producing *Salmonella* Dublin.

## Introduction

Antimicrobial resistance among enteric gram-negative bacteria represents an ongoing public health concern [1]. Non-typhoidal *Salmonella enterica*, historically associated with large outbreaks linked to dairy and agricultural practices, remains a significant cause of foodborne and zoonotic illness worldwide [2–4]. Antibiotic use in livestock production has contributed to the emergence and spread of resistant *Salmonella* strains capable of infecting humans [5, 6].

Although most non-typhoidal *Salmonella* infections manifest as self-limited gastroenteritis, dissemination to multiple organs and invasive disease in sterile sites can occur, particularly in individuals with underlying comorbidities or vascular abnormalities [7–10]. Treatment of invasive salmonellosis typically relies on third-generation cephalosporins or fluoroquinolones such as ceftriaxone or ciprofloxacin. However, increasing resistance to these agents has been documented worldwide [2, 8, 11, 12].

One mechanism of cephalosporin resistance involves plasmid-mediated AmpC (pAmpC) β-lactamases such as CMY-2, which confer resistance to many β-lactams including third-generation cephalosporins. Unlike extended-spectrum-beta-lactamases (ESBLs), CMY-2 and other AmpC enzymes can hydrolyze cephamycin antibiotics and are not inhibited by clavulanic acid. These enzymes are frequently carried on mobile genetic elements that facilitate horizontal gene transfer between Enterobacterales species [13–18].

We report a case of disseminated *Salmonella enterica* vascular graft infection with bacteremia, osteomyelitis, and involvement of the respiratory and urinary tracts. Whole-genome sequencing revealed a multidrug-resistant *Salmonella enterica* serovar Dublin isolate harboring a CMY-2–encoding plasmid and additional resistance determinants.

## Case

The patient is a 58-year-old male with a distal history of open aortic repair complicated by recent development of thoracoabdominal aortic aneurysm necessitating a thoracoabdominal branch endoprosthesis (TAMBE) who initially presented to an outside facility with complaint of subacute onset of abdominal pain radiating to his back associated with hemoptysis and melanotic stools. A mixed aneurysmal density at the thoracoabdominal junction concerning for hematoma with contained rupture was found on imaging. He most recently visited Mexico 3 months prior to this presentation, where he lived on a farm with animals including cows but stated that he did not interact with them. This does not exclude the possibility of exposure to contaminated animal products or to individuals or food handlers with direct animal contact. The patient was transferred to our facility and admitted to the surgical intensive care unit in septic shock. Blood, urine and bronchoalveolar lavage cultures grew *Salmonella*. A positron emission tomography scan demonstrated intense fluorodeoxyglucose activity at the location of the graft consistent with an infected endoprosthesis, while additional imaging showed thoracic osteomyelitis concerning for disseminated *Salmonella* infection. The patient was empirically started on piperacillin-tazobactam at the time of admission but subsequently changed to ertapenem due to concerns for possible ESBL production with ceftriaxone resistance on susceptibility testing. Gentamicin was also added initially for combination antibiotic therapy due to the initial persistence of bacteremia but discontinued after subsequent blood culture clearance on ertapenem. The patient ultimately was deemed not to be a surgical candidate due to the extent and size of the endoprosthesis, as well as not a candidate for outpatient parental antibiotic therapy due to an unstable housing situation. After goals of care discussion with the palliative care team, with the understanding that his infection was not curative without surgical intervention, the patient opted to leave on an indefinite course of oral sulfamethoxazole-trimethoprim.

## Methods

Whole-genome sequencing was performed using a hybrid approach. Short-read sequencing was conducted using the Illumina MiSeq (Illumina, San Diego, California) platform (v2 chemistry, 251 bp paired-end reads). Long-read sequencing was performed using Oxford Nanopore Technologies (Oxford, United Kingdom) sequencing with the SQK-RPB114.24 kit and super-accurate base calling.

Hybrid assembly was performed using Unicycler (v0.5.1, github.com/rrwick/unicycler). The assembled genome was annotated using Bakta (1.12.0, github.com/oschwengers/bakta). Sequences have been uploaded to NCBI database, PRJNA1458277. MOB-suite analysis (v3.1.9, github.com/phac-nml/mob-suite) was used to investigate plasmid context. Plasmids were visualized using Proksee (proksee.ca/, build 2026-02-25). Resistance genes were evaluated using CARD Nucleotide Database (3.2.6) and CLC Genomics (25.0.3).

## Results

Rapid molecular testing did not detect *bla*_CTX−M_, however a preliminary ceftriaxone disk diffusion was inoculated from the primary blood culture bottle and demonstrated resistance, raising concerns for an ESBL as mentioned in the case presentation. This prompted the switch to ertapenem for this patient. Once the organism was isolated, comprehensive phenotypic AST (Table 1) was completed 1–2 days later and no clavulanic acid effect was observed, shifting the suspicion from ESBL to an AmpC β-lactamase. Given this AST profile, genomic analysis was performed to identify any insidious resistance determinants, revealing the following antimicrobial resistance genes (Table 2): Hybrid assembly of the genome revealed that the isolate was most closely related to *Salmonella enterica* subsp. *enterica* serovar Dublin (NZ_CP032449). Additionally, genomic analysis identified a D87N mutation in *gyrA*, which has been associated with reduced susceptibility to fluoroquinolones through alterations in the quinolone resistance-determining region of DNA gyrase [19, 20]. No plasmid-mediated quinolone resistance determinants (*qnr*,* qepA*, or *oqxAB*) were found in our annotation and *parC* did not contain mutations known to cause fluoroquinolone resistance.

**Table 1:** Phenotypic susceptibility testing performed on the *Salmonella* isolated from the respiratory tract of the patient

Antibiotic Class	Antibiotic	MIC / Value	Interpretation
Penicillins / β-lactamase inhibitor	Amoxicillin/Clavulanate	> 32	Resistant
	Piperacillin/Tazobactam	≤ 8	Susceptible
Cephalosporins / β-lactamase inhibitor	Cefazolin	> 32	Resistant
	Ceftriaxone	64	Resistant
	Ceftazidime	32	Resistant
	Cefepime	≤ 0.5	Susceptible
	Ceftazidime/Avibactam	≤ 2	Susceptible
Carbapenems / β-lactamase inhibitor	Meropenem	≤ 0.25	Susceptible
	Imipenem	≤ 1	Susceptible
	Ertapenem	≤ 0.25	Susceptible
	Meropenem/Vaborbactam	≤ 0.06	Susceptible
Fluoroquinolones	Ciprofloxacin	0.25	Intermediate
	Levofloxacin	0.5	Susceptible
	Moxifloxacin	1	—
Tetracycline class	Minocycline	2	Susceptible
	Tigecycline	0.5	Susceptible
	Omadacycline	2	—
	Eravacycline	0.25	—
Others	Azithromycin	4	—
	Trimethoprim/Sulfamethoxazole	≤ 1/20	Susceptible

Microbroth dilution was performed and interpreted according to CLSI standards

**Table 2:** Genotypic resistome for the *Salmonella* isolate in this study

Antibiotic Class	Gene	Resistance Mechanism
β-lactams	CMY-2	Class-C β-lactamase
	TEM-206	Class-A β-lactamase
Aminoglycosides	AAC(6’)-Iy	Modifying enzyme
	APH(6)-Id	Modifying enzyme
	APH(3’’)-Ib	Modifying enzyme
	APH(3’)-Ia	Modifying enzyme
Fluoroquinolones / efflux regulation	golS	Efflux pump regulator
	gyrA [D87N]	Target protein mutation
Tetracyclines	tet(A)	Efflux pump
Phenicols	floR	Efflux pump
Sulfonamides	sul2	Target replacement protein
Multidrug efflux systems	mdsA	Efflux pump
	mdsB	Efflux pump
	mdsC	Efflux pump
	sdiA	Efflux pump regulator
	golS	Efflux pump regulator

**Fig. 1:**
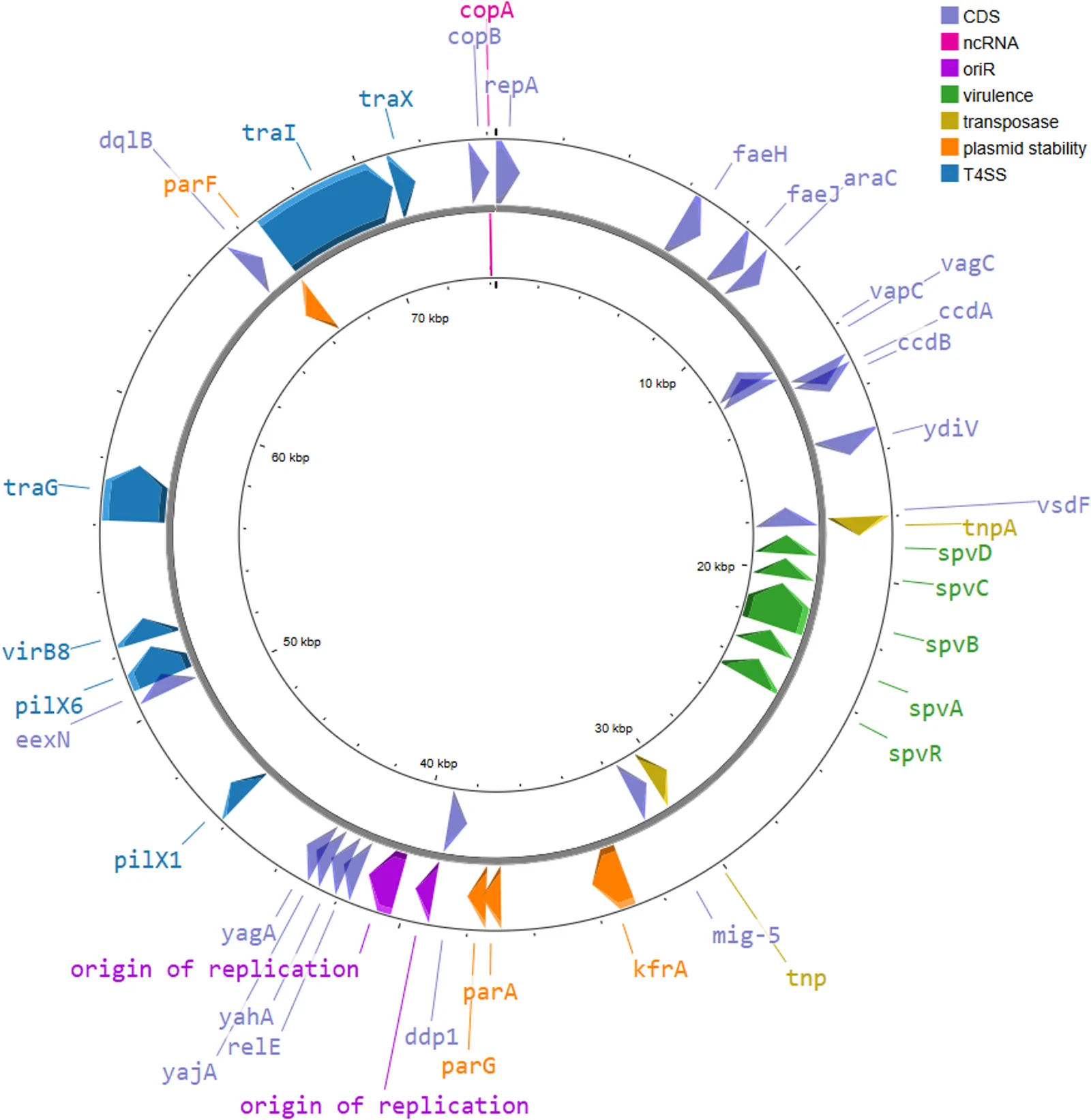
IncFII(S)/IncX1 virulence plasmid identified in this isolate. Over 98% pairwise identity was found between the plasmid shown and the reference (CP117375)

**Fig. 2:**
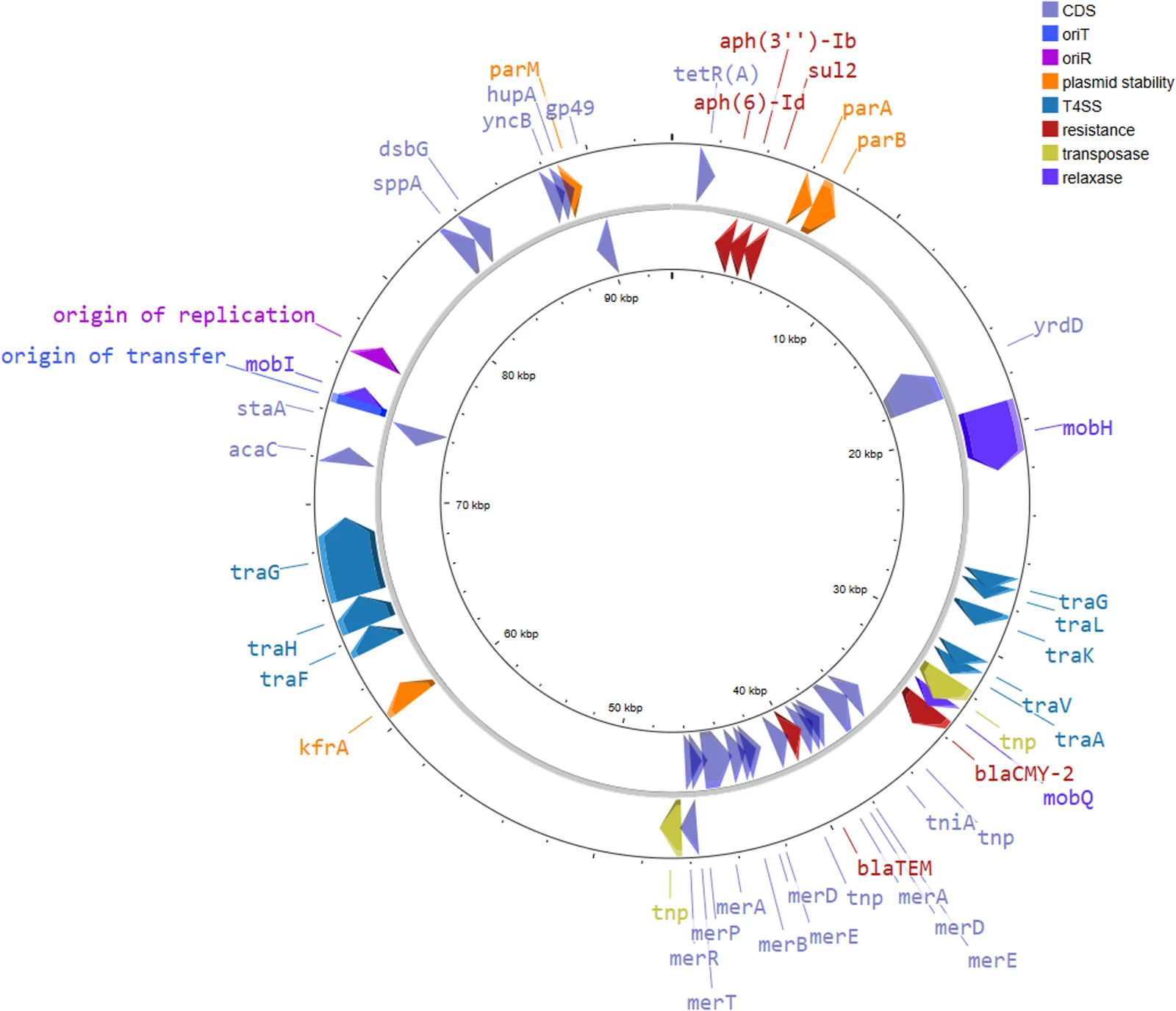
Multidrug-resistant IncA/C2 plasmid identified in this isolate. This plasmid most closely related to and was only missing 8.5 kb from the reference (CP063757) but otherwise showed over 98% pairwise identity

Two major plasmids were identified in the assembled genome. First, a ~ 74 kb multireplicon IncFII/IncX1 virulence plasmid carrying conjugation associated genes (*tra*/*pilX*/*virB*), plasmid stability determinants (*par*/*kfr*), and the *spvRABCD* operon associated with increased virulence and systemic dissemination in *Salmonella* (Fig. 1) [21, 22]. Next identified was a ~ 94 kb resistance plasmid containing *bla*_CMY−2_, *bla*_TEM_ and *sul2* genes (Fig. 2). Plasmid typing predicted a conjugative IncA/C2(IncC) replicon on the contig containing *bla*_CMY−2_. The *bla*_CMY−2_ gene was located within a plasmid mobility region containing a MobQ relaxase, a downstream *blc*-like lipocalin gene, and an adjacent IS6-family transposase (IS15DIV), consistent with an IS-rearranged CMY-2 resistance module.

## Discussion

Disseminated non-typhoidal *Salmonella* disease has been associated with certain host risk factors as well as specific *Salmonella* serovars, including *Salmonella enterica* serovar Dublin, which is known to cause systemic infections more frequently than many other non-typhoidal serovars [7, 23–25].The isolate described in this report carried multiple antimicrobial resistance determinants, including a plasmid-encoded CMY-2 AmpC β-lactamase. These enzymes confer resistance to third-generation cephalosporins and are not inhibited by β-lactamase inhibitors such as clavulanic acid, explaining the absence of a clavulanic acid effect during phenotypic susceptibility testing [7, 26, 27]. On the same plasmid, the isolate carried sulfonamide resistance determinant *sul2*, and *bla*_TEM−206_, a TEM-family β-lactamase gene that may also contribute to β-lactam resistance seen in this isolate [28, 29].

Although pAmpC genes increasingly are reported in other Enterobacterales including *Escherichia coli*, *Klebsiella pneumoniae*, and *Shigella sonnei* at our institution, pAmpC *Salmonella* remains rarely reported in the U.S. clinical setting [18–20, 24]. CMY-type AmpC enzymes, particularly *bla*_CMY−2_, have been identified in *Salmonella* from agricultural surveillance and human outbreak investigations and are often associated with mobile plasmids such as IncC, supporting ongoing horizontal transfer and highlighting their clinical significance in invasive disease [33, 34]. In a comparative genomic study of 69 U.S. *Salmonella* Dublin isolates, *bla*_CMY−2_ was detected on multidrug resistance/virulence plasmids, with only eight human isolates included, further underscoring that pAmpC *Salmonella* infection in humans remains uncommon but is an important zoonotic hazard, particularly in the setting of agricultural exposure and plasmid-mediated dissemination [30].

Most current commercial blood molecular platforms are unable to identify pAmpC and while disk diffusion methods (clavulanic acid, cefoxitin synergy) can help point to a mechanism, no reliable AST profile definitively distinguishes AmpC versus ESBL producers in the clinical lab. Further, pAmpC production in combination with porin loss can lead to carbapenem resistance during therapy, limiting treatment options [23, 31]. While porin loss was not seen in this case, these realities present a challenge in diagnosing resistance in *Salmonella* bacteremia and may delay treatment optimization.

In our case, the patient was initially treated with Pip/Tazo and transitioned to ertapenem following preliminary AST results demonstrating β-lactam resistance in the absence of a CTX-M target, raising concern for non-CTX-M-mediated resistance, such as AmpC or an alternate ESBL. While carbapenems recommended by IDSA [32] as first-line therapy for invasive ESBL-producing Enterobacterales infections, they are also generally stable to AmpC hydrolysis and represent a reliable treatment option when there is diagnostic uncertainty. Cefepime may remain susceptible in CMY-2–producing Enterobacterales because it is relatively stable to AmpC hydrolysis; however, susceptibility can vary with enzyme expression and testing conditions. Further, cefepime is less reliable in the setting of potential ESBL co-production in AmpC-producing Enterobacterales (AmpC-E). Additionally, β-lactam/β-lactamase inhibitor combinations such as piperacillin-tazobactam may be suboptimal due to limited activity of tazobactam against AmpC enzymes [26]. Given the severity of infection, evidence of dissemination, and incomplete early genotypic characterization, escalation to ertapenem was undertaken to ensure adequate coverage pending definitive susceptibility and genomic data. This approach is consistent with current guidance prioritizing carbapenems in serious invasive infections when resistance mechanisms are uncertain.

Hybrid genome sequencing identified a virulence plasmid containing *spv* genes, which have been shown to enhance *Salmonella* intracellular replication and systemic dissemination [22, 33]. The coexistence of virulence plasmids and antimicrobial resistance plasmids raises concern for the emergence of strains capable of causing severe invasive disease with limited therapeutic options.

In addition to plasmid-mediated resistance mechanisms, the isolate harbored a D87N mutation in *gyrA*, which affects the quinolone resistance-determining region of DNA gyrase and contributes to reduced fluoroquinolone susceptibility [23, 34]. This finding is consistent with the intermediate ciprofloxacin susceptibility observed in phenotypic testing.

This case also illustrates the propensity of *Salmonella* to disseminate beyond the gastrointestinal tract, particularly in the presence of vascular abnormalities or prosthetic material [10, 35]. The patient developed bacteremia with involvement of the thoracic skeleton and respiratory tract in the setting of an infected vascular endograft. Osteomyelitis and vascular infections are recognized complications of invasive *Salmonella* infection [36, 37].

Finally, genomic analysis identified the presence of *sul2* on the CMY-2-encoding plasmid, which may confer resistance to sulfonamides [38–40]. Trimethoprim resistance is usually mediated by *dfrA*-family genes, which was not found in this isolate. Others have noted the presence of *sul2* in Bactrim-susceptible *Salmonella* isolates, while true Bactrim-resistant isolates also harbored a *dfrA* gene [41]. Although oral step-down therapy with trimethoprim-sulfamethoxazole can be appropriate for AmpC-E when susceptibility is demonstrated, consistent with IDSA guidance, the detection of *sul2* in this isolate raised concern for potential compromise of the sulfamethoxazole component. Although there are no interpretative criteria available for azithromycin against non-typhoidal *Salmonella*, given an MIC of 4, an epidemiological cutoff value (ECV) of 16, and the absence of macrolide resistance genes, azithromycin was recommended as a potential oral therapeutic option.

## Conclusions

This case describes a disseminated infection caused by multidrug-resistant *Salmonella enterica* serovar Dublin harboring a CMY-2-encoding plasmid and multiple additional resistance determinants. Hybrid whole-genome sequencing revealed both a virulence plasmid containing *spv* genes and a multi-drug resistance plasmid carrying, highlighting the role of mobile genetic elements in the evolution of invasive and drug-resistant *Salmonella* strains.

The presence of osteomyelitis and respiratory involvement underscores the ability of *Salmonella* to disseminate systemically, particularly in the presence of vascular prosthetic infection. Continued genomic surveillance and integration of sequencing data into clinical microbiology workflows are essential for identifying emerging resistance mechanisms and informing treatment decisions for invasive *Salmonella* infections. This case demonstrates the interconnected human, animal, and environmental drivers of antimicrobial resistance, emphasizing the need for a One Health approach to surveillance and management. Clinicians should be aware of the emerging drug resistance mechanism with pAmpC *Salmonella* and adjust treatment based on phenotypic and genotypic profiles.

## Data Availability

The sequences of the chromosome and plasmids described in this study were deposited to the NCBI GenBank (PRJNA1458277).
